# Training and National deficit of psychiatrists in India – A critical analysis

**DOI:** 10.4103/0019-5545.69218

**Published:** 2010-01

**Authors:** M. Thirunavukarasu, P. Thirunavukarasu

**Affiliations:** Department of Psychiatry, Stanley Medical College, Member of Senate and President Faculty of Medical Specialties, The Tamil Nadu Dr. MGR Medical University, Chennai, India; 1 University of Pittsburgh Medical Center, USA

**Keywords:** Burden of mental illness, deficit of psychiatrists, psychiatric training

## Abstract

India is the second most populous country in the world, with an estimated current population of 1.17 billion. This article aims to estimate the deficit of psychiatrists in India in relation to epidemiological burden of mental illness, propose short-term and long-term strategies to tackle the deficit and emphasize the importance of modifying the curriculum of undergraduate medical education to enable the proposed strategies. With 6.5% prevalence of serious mental disorder, the average national deficit of India is estimated to be 77%. More than one-third of the population has more than 90% deficit of psychiatrists. The authors estimated that the undergraduate medical curriculum devotes only 1.4% of lecture time and 3.8-4.1% of internship time to psychiatry, thereby leaving the general practitioners and the non-psychiatrist specialists unprepared to competently deal with mental illness in their practice. We propose short and long-term strategies to manage this deficit of psychiatrists.

## INTRODUCTION

“*A chain is only as strong as its weakest link*”

English Proverb

Implicatively, the strength of the mental healthcare in India is a function, not of the quality and competence of the specialist (i.e. the psychiatrist), but the quality and competence of the generalist. Maximum improvement in mental healthcare in India will be realized only by training the non-psychiatrist healthcare providers, who work in the largest catchment area of mental illness.

## THE BURDEN

The burden of mental illness in India is enormous. As per the Government of India’s National Commission on Macroeconomics and Health Report of 2005, the prevalence of ‘*serious*’ mental illness in the Indian population is at least 6.5%,[1] which by rough estimate would be 71 million people; the latter is larger than the last released census population[2] of entire Tamil Nadu (~62 million) or Madhya Pradesh (~60 million) or Rajasthan (~56 million) or Karnataka (~52 million) or Gujarat (~50 million) or Orissa (~36 million) or any of most of the states of the Indian nation. In essence, if all the people with serious mental disorder were to be institutionalized for treatment or rehabilitation, we would in reality need almost another state!

There may be several medical illnesses that may have prevalence higher than 6.5%, but that the prevalence of only the ‘*serious*’ form of a certain medical illness amounting to that high number is unnerving. Not only is the magnitude of the burden from mental illnesses high, but the nature of the impact produced by mental illness to the individual and society is also far more widespread and consequential than most other medical diagnoses. In India, mental health disorders cause more loss of Disability Adjusted Life Years (DALYs) than even some of the very common diagnoses including cancer, diabetes, tuberculosis, HIV/AIDS and malaria, all of which receive more attention from the medical community than mental health disorders. Mental health disorders alone account for about 25%[1] of total DALYs lost due to priority non-communicable diseases. Mental illness results in more DALYs lost than some of the very common diagnoses in India, as shown in Table 1.

**Table 1:** A partial list of conditions that result in less disability adjusted life years lost, compared to mental health disorders

Communicable diseases	Non communicable diseases
Tuberculosis	Cancers
HIV/AIDS	Diabetes
Diarrheal diseases	Blindness
Leprosy	COPD/asthma
Malaria and other vector-borne diseases	Oral diseases
Childhood communicable diseases	

## THE DEFICIT

**Figure 1:**
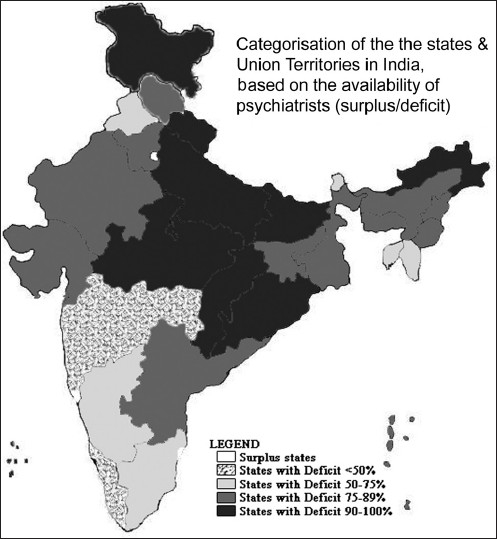
Indian map showing state-wise psychiatrist deficit/surplus distribution

While the burden is in catastrophic proportions, the soul annihilating issue is the deficit of psychiatrists. Using the data sourced from National Survey of Mental Health Resources[1] carried out by the Directorate General of Health Services in 2002, we calculated the estimated deficit of psychiatrists in India, based on the available number of psychiatrists and the ideal number required (~1.0 per 100,000 population). We further categorized the states and union territories (UT) based on the amount of deficit of psychiatrists, as seen in Table 2 and Figure 1.

**Table 2:** Categorization of the states and union territories in India; based on availability (surplus/deficit) of psychiatrists

Classification by deficit/surplus of psychiatrist	State	Surplus/ deficit (%)
Surplus states	Chandigarh	244.00
	Goa	86.00
	Pondicherry	50.00
	Delhi	13.00
States with deficit <50%	Kerala	25.16
	Maharashtra	49.74
States with deficit 50-74%	Mizoram	55.56
	Tamil Nadu	57.81
	Sikkim	60.00
	Karnataka	62.43
	Punjab	63.22
	Tripura	70.97
States with deficit 75-89%	Andaman and Nicobar	75.00
	Daman and Diu, Dadra and Nagar Haveli	75.00
	Manipur	75.00
	Nagaland	75.00
	Andhra Pradesh	76.22
	India	77.64
	Meghalaya	78.26
	Gujarat	80.79
	Haryana	81.43
	Jharkhand	81.48
	Himachal Pradesh	86.89
	Rajasthan	86.73
	Assam	89.10
	West Bengal	89.65
States with deficit 90-100%	Arunachal Pradesh	90.00
	Chhattisgarh	92.75
	Uttaranchal	92.86
	Uttar Pradesh	93.07
	Orissa	94.82
	Jammu and Kashmir	96.00
	Bihar	96.62
	Madhya Pradesh	98.01
	Lakshadweep	100.00

As often is the case with India, there was a huge diversity across the Indian terrain, even in the deficit of psychiatrists. On one hand, four states, namely, Chandigarh, Delhi, Goa and Pondicherry had a surplus of psychiatrists, ranging from 244% surplus in Chandigarh to a 13% surplus in Pondicherry. In all the other states/UTs, there was a deficit, with only two states having a deficit of less than 50%. Nine states had more than 90% deficit. As per our estimation from the 2001 Census, these nine states amounted to 37.6% of the Indian population and 41.1% of the rural population of India. Lakshdweep had a 100% deficit, implying that more than 60,000 Indians living nearly 200-300 km off the west coast of the subcontinent had no psychiatrists.

We calculated the average national deficit of psychiatrists to be 77% and 17 states/UTs were below this average. It is to be duly noted that most of the above estimates were made from the census data from 2001, and the situation now in 2010 is probably far worse, given the continuing expansion in population and nil perceivable expansion in psychiatrist output in the last decade.

## THE PLIGHT

In India, such a deficit of specialists may be present in other fields of medicine too. But our model of medial education usually serves to mitigate such deficit. Indian undergraduate medical education is so well rounded that competent general practitioners and internists effectively compensate for a dearth of specialists. For example, there may be a big deficit of cardiologists in India, but the average internist is well trained to effectively diagnose and treat cardiologic conditions. They are also prudent enough to refer patients to a higher level of care when needed. Our model of medical education has produced doctors who can treat a variety of conditions, including uncomplicated child-births, infectious diseases, pediatric conditions, some common ophthalmological and ENT conditions, skin disorders, etc. Unfortunately, in the case of psychiatry, our model of undergraduate education, one of the best known, is just not good enough.

**Figure 2:**
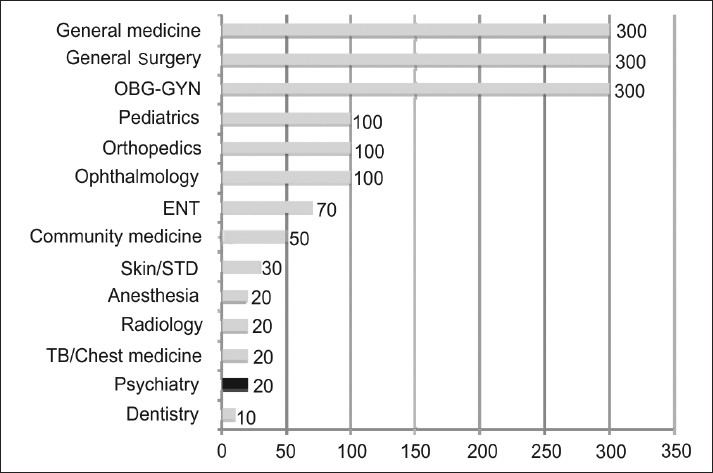
Demonstrates the number of hours of lecturing or clinical instruction devoted to the various specialties during MBBS, as stipulated by the Medical Council of India. The data plotted in the graph was obtained by accessing the official website of the Medical Council of India (www.mciiindia.org, last accessed December, 2009)

**Figure 3:**
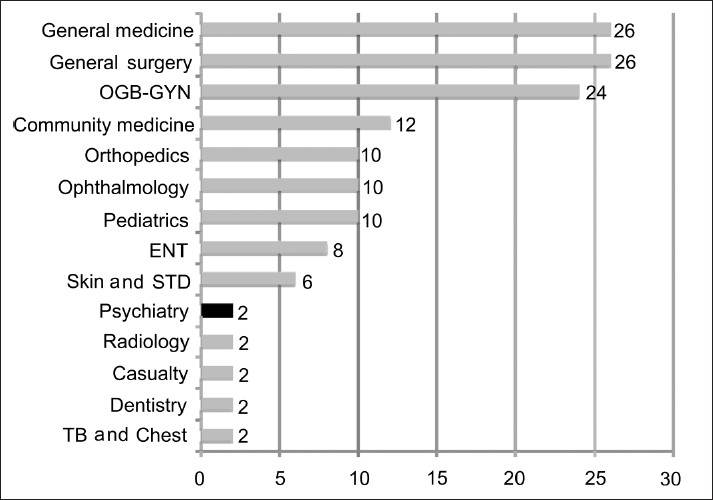
Demonstrates the number of weeks of clinical internship experience devoted to the various specialties during MBBS, as stipulated by the Medical Council of India. The data plotted in the graph was obtained by accessing the official website of the Medical Council of India (www.mciiindia. org, last accessed December, 2009)

Our undergraduate education does not prepare our future generation of doctors to handle the herculean burden of psychiatric illnesses. This is a direct result of the miniscule amount of teaching and clinical experience in psychiatry, as stipulated by the curriculum designed by the Medical Council of India. Psychiatry, as a subject of study has been offered a disproportionately small amount of time during the undergraduate course, as seen in Figures 2 and 3

Psychiatry, as a subject of study, is offered only a minimum of 20 hours of clinical lecturing.[3] This constitutes to only 1.4% of the total amount of lecturing hours. When realistic aspects of lecturing are taken into account, this miniscule amount of time devoted to psychiatry seems to appear more like a ritual for the achievement of political correctness in adequacy of training, than an honest willful attempt to educate the medical students.

Philosophically, the lack of knowledge in medicine or the attrition of gained knowledge is pardonable, but the lack of skill is not. For example, every doctor is not expected to always possess full knowledge of the physiological aspects of anemia or jaundice, but he/she is expected always to effectively diagnose them and act accordingly. Unfortunately, in psychiatry, our MBBS graduates are not only insufficiently imparted with knowledge, but also suffer from a profound lack of the basic psychiatric skills to not only confront psychiatric diseases, but even to be able to pick up psychiatric manifestations of physical disease and the physical manifestations of psychiatric disease. This is because of the lack of sufficient clinical work experience during internship. As per the regulations of Graduate Medical Education of 1997,[3] psychiatry experience during house internship for MBBS students was a part of the parent general medical rotation, wherein the students were exposed to an exclusive psychiatric rotation, only as deemed fit by the department of medicine or the institution. In essence, psychiatry was not mandatory for the successful completion of house internship. However, in the latter half of 2008, MCI released its ‘*Regulations on Graduate Medical Education (Amendment), 2008*’, wherein some changes were made to the distribution of times allocated to different specialties for the interns.[3] They can be summarized as shown in Table 3.

**Table 3:** Summary of time distribution to various specialties for house interns for the completion of MBBS. The table was derived from the data obtained from the official website of the Medical Council of India (www.mciindia.org, last accessed in December, 2008)

Specialty	MCI stipulated internship time in months		Effect
	Before 2008	After 2008	
Community medicine	3	2	Decreased
General medicine	2	1.5	Decreased
General surgery	1.5	1.5	No change
OBG-GYN/Family welfare	2	2	No change
Pediatrics	0.5	1	Increased
Orthopedics/PMR	0.5	1	Increased
Ophthalmology	0.5	0.5	No change
ENT	0.5	0.5	No change
Elective rotation	0.5	0.5	No change
Casualty/Emergency medicine	1	0.5	Decreased
Psychiatry	Optional in electives	0.5	Made compulsory
Anesthesia	Optional during surgery rotation	0.5	Made compulsory

Hence, by the amendment of 2008, interns need to rotate exclusively through psychiatry for two weeks. Also, psychiatry is offered as one of the options for the elective rotation available for interns for two weeks. The authors, as would many psychiatrists, welcome this change; however, this is still not good enough. This time constitutes about 3.8-4.1% of the total internship time of 365 days or 12 months, depending on administrative situations. Two weeks, the authors contend to be disproportionately less for the attainment of sufficient skills in psychiatry [Figure 3]. Two weeks of clinical experience is in reality a minimum of 10 working days, wherein administrative duties, orientation and acclimatization would by themselves consume the intern and rob him of fruitful applicable experience.

## CONFRONTING THE PROBLEM

The crux of the problem here is that our model of medical education and training is not adequate to meet the demands of the rising burden. The psychiatrist community is aware of the problem, but the problem has not been addressed seriously. Table 4 is a list of publications in the Indian Journal of Psychiatry in the last few decades that have addressed the issue of training in psychiatry.

**Table 4:** List of publications in Indian Journal of Psychiatry in the last 25 years (1984-2008), pertaining to training in psychiatry

Author	Title	Year	Target trainees	Type of article
S. D. Sharma[4]	General hospital psychiatry and undergraduate medical education	1984	Medical students	Editorial
P. Kulhara[5]	General hospitals in postgraduate psychiatric training and research	1984	PG/Residents	Communication
Shiv Gautam[6]	Development and evaluation of training programs for primary mental health care	1985	General practitioners	Original article
Rajeev Gupta *et al.*[7]	Psychiatric training and its practice: A survey of 86 practitioners	1987	General practitioners	Original article
K. Praveenlal *et al.*[8]	Capitals not needed	1988	Medical students	Original article
C. Shamasundar *et al.*[9]	Training general practitioners in psychiatry - A new venture	1988	General practitioners	Original article
C. Shamasundar *et al.*[10]	Clinical vignettes for assessment of training general practitioners in psychiatry	1989	General practitioners	Original article
C. Shamasundar *et al.*[11]	Training general practitioners in psychiatry - An ICMR multi-center study	1989	General practitioners	Original article
K. Bhaskaran[12]	Undergraduate training in psychiatry and behavioral sciences - the need to train the trainers	1990	Medical students	Editorial
Anna Tharayan *et al.*[13]	Undergraduate training in psychiatry. An evaluation	1992	Medical students	Original article
Satyavati Devi[14]	Short term training of medical officers in mental health	1993	General practitioners	Original article
K. Kuruvila[15]	A common minimum program needed in post-graduate training in Psychiatry	1996	PG/Residents	Editorial
J.K Trivedi[16]	Importance of undergraduate psychiatric training	1998	Medical students	Editorial
K. Kuruvila[17]	The future of psychiatry: The need to return to the field of medicine	1998	Medical students	Presidential address
R.K Chadda *et al.*[18]	Awareness about psychiatry in undergraduate medical students in Nepal	1999	Medical students	Original article
C. Shamasundar[19]	“Whither training in psychiatry and psychosomatic medicine!” What need to be done?	2004	Medical students	Communication
Indla Ramasubba Reddy[20]	Undergraduate psychiatry education: Present scenario in India	2007	Medical students	Communication
A.B.Ghosh *et al.*[21]	Why should psychiatry be included as examination subject in undergraduate curriculum?	2007	Medical students	Communication
R. Srinivasa Murthy *et al.*[22]	Undergraduate training in psychiatry: World perspective	2007	Medical students	Communication
M. Thirunavukarasu[23]	Psychiatry in UG curriculum of medicine: Need of the hour	2007	Medical students	Communication

It is notable from the list of publications that 90% of them (18 out of 20 publications) were aimed at training medical students and/or general practitioners. From this observation, it is probably safe to assume that most of the academia of the Indian psychiatrist community is almost unanimous in the idea that the training of non-psychiatrists is at least as important, if not more, than the training of psychiatrists with respect to dealing with the burden of psychiatric illness in the community. However, only 45% of these publications (9 out of 20 publications) were original articles. The rest were monologues, communications or editorials where its authors were simply blowing off their steam. This also means that there needs to be more objective evidence to support the claims of ineffective training among medical students. Except for the National Mental Health Program (NMHP) and a few isolated task force-propelled training activities of general practitioners, less has been achieved in terms of concrete action.

## SHORT-TERM STRATEGY

**Figure 4:**
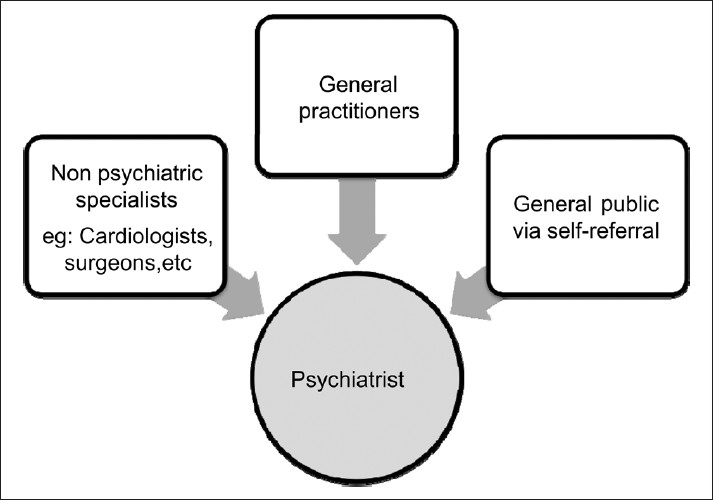
Dynamics of patient flow leading to specialist psychiatric care

It is important to understand the dynamics of patient accrual for treatment, in order to tackle this problem in the short term. The authors would like to approach the current issue from the patient’s perspective. Patients with mental disorders (with or without other co-morbid conditions) who seek medical care end up in the hands of one of the three following groups of physicians [Figure 4].

1. Psychiatrists (through self referral)
2. General Practitioners (GPs)
3. Other specialists

Those who seek the psychiatrist’s help are cared for accordingly. Those who end up in the care of GPs or non- psychiatric specialists may not, in most cases, receive adequate attention to their mental disorder. The patients need to get diagnosed and appropriately referred to the psychiatrist at this stage. This can be achieved in one of two ways:

- Training the practicing GPs and specialists in basic psychiatric skills necessary to identify mental health disorders and refer them for specialist care.
- Introducing strict policies wherein the GP/specialist must document presence or absence of psychiatric symptoms, which will lead to mandatory notification of the patient and/or referral to a psychiatrist to ensure that appropriate care is available.

The comparative advantages and disadvantages of these two approaches are summarized in Table 5.

**Table 5:** Comparison of the suggested short-term measures to mitigate the burden undiagnosed and untreated mental illness

Strategy	Advantages	Disadvantages
Training of practicing doctors (GPs and nonpsychiatric specialists)	Does not require legislation Allows interaction with physicians	Requires organizational support Requires large funds to conduct these sessions
		Attendance may be poor
		The prime target trainees (those with high volume practice) may be especially low in attendance
		Effect may not seen quickly
Strict Policy decisions for routinely documenting psychiatric symptoms, which will lead to mandatory notification to patient and/or specialist referral	Less expensive in the long run Very effective in the short term May prevent grossly missed psychiatric mortality or morbidity such as suicide, homicide, etc	Such policies may meet with friction amongst the specialists before its widely accepted Requires legislation

## LONG-TERM STRATEGY

The only long-term solution to this problem is to train undergraduate medical students to provide a strong fundamental basis in psychiatry, so that they are trained to routinely look for psychiatric illness in all the patients that they care for. However, this would mean a basic architectural reform in the curriculum of medical education to include all of the following:

1. Education in the basic fields required to learn psychiatry such as psychology, behavioral sciences, sociology, psychopharmacology, etc.
2. Clinical Psychiatry rotations with proportionate time allocations.
3. Exclusive examination (theory and clinical) to assess achievement of learning objectives.
4. Exclusive clinical internship experience (out-patient and in-patient) for the successful completion of MBBS.

## THE BEST STRATEGY

The authors strongly feel that the best strategy to tackle this problem of huge burden and deplorable deficit is to actually train the general public! We feel that increasing awareness about mental illness, removing the myths and misconceptions of mental disorders and educating the general public of the scope and importance of mental health care will probably work more quickly, more effectively and will be long lasting. This could mean educating school children about the basics of mental health and well being, just like teaching them about the basics of hygiene and physical health. In the authors’ opinion, if this approach is followed, this might prevent stigmata and misconceptions of mental illness in future and to our surprise may even become non-existent in the next generation. This could also prevent problems in childhood, which if left unattended could lead to more embarrassing situations where we might need to implement measures like sex education, drug abuse and alcohol education, behavior modification, etc among school children. While the latter may well be effective, it just seems more intelligent and wise to teach the basics of mental health to children (and parents and teachers) which might prevent the need for such problem-specific interventions.

## SUMMARY

The burden of mental illness is in himalayan proportions and the deficit of psychiatrists is large, with an average national deficit of 77%. The problem is expected to increase in an unabated fashion and we are clearly not prepared to deal with the situation. The long-term solution would be a strong training in psychiatry at the undergraduate level. However, it could take several decades to see the effect of such a measure. The short-term solutions would include either or both of two measures, namely the training of practicing non-psychiatrist physicians and the implementation of strict policies to enforce guideline-oriented examination of all patients to look for psychiatric problems leading to mandatory patient notification and/or psychiatrist referral. The best, most effective, fool-proof and long-lasting solution, in the authors’ opinion, would be the education of the general public.
